# *In vivo* selection of CMY-219 conferring resistance to ceftazidime-avibactam in an OXA-484-producing *E. coli* ST410

**DOI:** 10.1128/aac.01335-25

**Published:** 2026-02-12

**Authors:** Agnès B. Jousset, Delphine Girlich, Saoussen Oueslati, Aurélien Birer, Anne Delaval, Caroline Guyot, Ines Rezzoug, Cécile Emeraud, Thierry Naas, Rémy A. Bonnin, Laurent Dortet

**Affiliations:** 1 Center for Immunology of Viral, Auto-immune, Hematological and Bacterial Diseases (IMVA-HB/IDMIT/UMRS1184), Université Paris-Saclay, Inserm, CEA 27048, Fontenay-aux-Roses & Le Kremlin-Bicêtre, France; 2 Department of Bacteriology-Hygiene, Bicêtre Hospital, Assistance Publique-Hôpitaux de Paris 26930, Le Kremlin-Bicêtre, France; 3 French Associated National Reference Center for Antibiotic Resistance, Le Kremlin-Bicêtre, France; 4 SEPSIS Comprehensive Center–IHU SEPSIS, Le Kremlin-Bicêtre, France; 5 Service de Bactériologie, Groupe Hospitalier Intercommunal du Raincy-Montfermeil 37063, Montfermeil, France; University of Pennsylvania Perelman School of Medicine, Philadelphia, Pennsylvania, USA

**Keywords:** CMY variants, ST410, ceftazidime-avibactam, *E. coli*

## Abstract

Resistance to ceftazidime-avibactam (CAZ-AVI) is a growing problem. This study describes the selection of CMY-219, a CMY-42 variant (G156D), conferring resistance to CAZ-AVI in an OXA-484-producing *Escherichia coli* ST410 after treatment. It raises concern about the risk of selection of CMY variants under CAZ-AVI exposure in ST410 and related clones, which commonly carry CMY-42, are prone to carbapenemase acquisition, and harbor modified PBP3.

## INTRODUCTION

Avibactam is the first non-β-lactam β-lactamase inhibitor active against ESBLs, AmpC, and serine carbapenemases. Accordingly, ceftazidime-avibactam (CAZ-AVI) is now considered as the reference for the treatment of infections caused by KPC and OXA-48 producers (1, 2). CAZ-AVI has demonstrated *in vitro* and clinical efficacy against carbapenemase-producing Enterobacterales, except against metallo-β-lactamase producers (3–5).

Resistance to CAZ-AVI mainly arises through increased expression and/or active-site mutations in class A β-lactamases (notably KPC) (6), often combined with reduced permeability, efflux upregulation, or PBP3 alterations (7–9). Few studies described mutations within CTX-M-15 or chromosomal AmpC in Enterobacterales, whether exposed *in vitro* to CAZ-AVI in *Citrobacter freundii*, *Enterobacter cloacae* (10), or *in vivo* after a patient’s exposure to *Klebsiella pneumoniae* and *Klebsiella aerogenes* (11, 12). Four CMY variants responsible for CAZ-AVI resistance have been identified in clinical isolates: CMY-178, CMY-185, and CMY-192 in *Escherichia coli* and CMY-172 in *K. pneumoniae* (13–16). These variants differ from CMY-2 by at least four amino acid substitutions or indels (16). CMY-42, a V231S CMY-2 variant, showed increased hydrolysis of ceftazidime and aztreonam (17). Here, we describe CMY-219, a single variant of CMY-42, selected after CAZ-AVI treatment in *E. coli* ST410.

Three clinical *E. coli* isolates (EC1-399F8, EC2-459F3, and EC3-511J2) from the same patient were submitted to the French National Reference Center (F-NRC) for Antimicrobial Resistance for investigation regarding carbapenemase production. MICs were determined by broth microdilution using customized Sensititre plates (Thermo Scientific, Les Ulis, France) and gradient strip (Liofilchem) for compounds not included or to extend the concentration range. Cefiderocol testing used the UMIC Cefiderocol (Bruker, Bremen, Germany). Results were interpreted using EUCAST guidelines 2025. Whole-genome sequencing was performed on a NextSeq 500 Illumina System. Assemblies were generated with Shovill v.1.1.0 and SPAdes v.3.14.0 and were analyzed for resistome, sequence type, and plasmid content on the CGE online platform (https://www.genomicepidemiology.org/). Genomic data are available under BioProject number PRJNA1279145.

**Fig 1:**
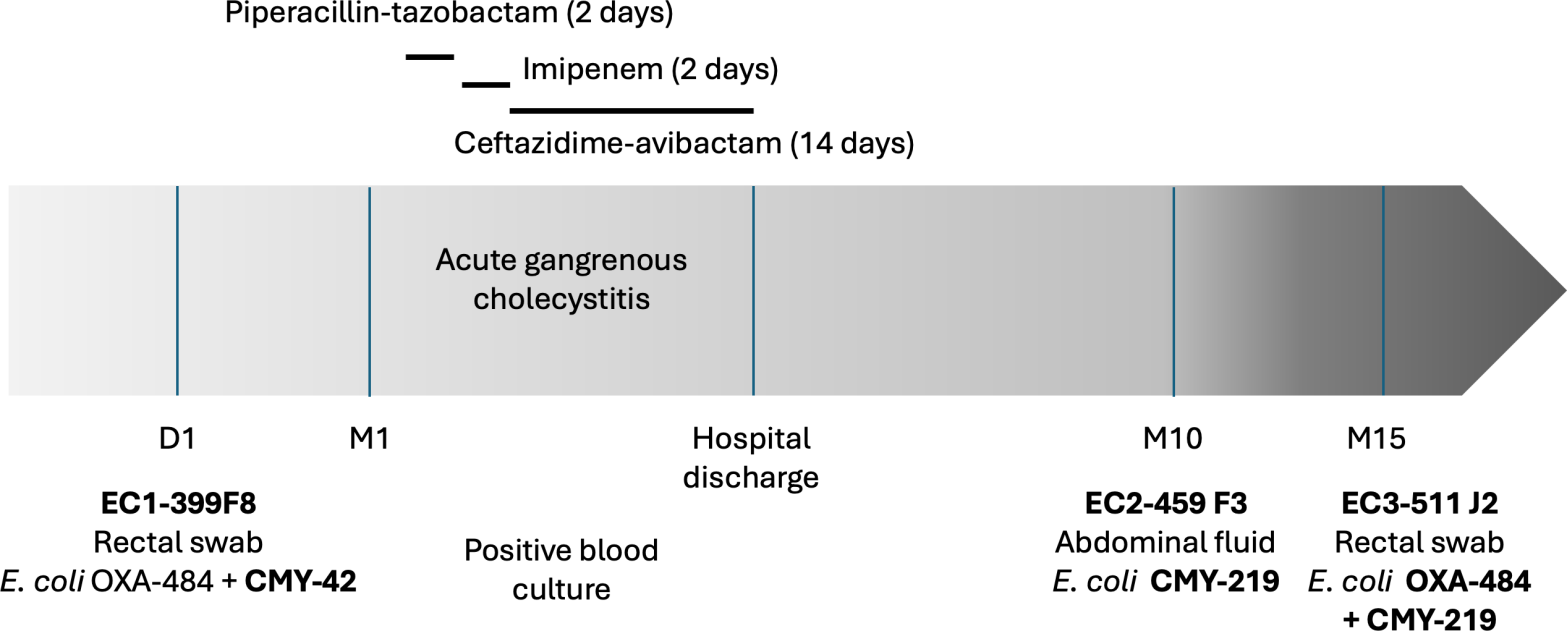
Timeline of clinical events, antimicrobial treatments, and *E. coli* isolate collection in a patient treated with ceftazidime-avibactam for 14 days.

EC1-399F8 was isolated at day 1 from a rectal swab of a patient recently diagnosed with pancreatic adenocarcinoma. This *E. coli* isolate produced an OXA-484 carbapenemase, along with CMY-42 and TEM-1 β-lactamases. This isolate was susceptible to CAZ-AVI (MIC = 1 mg/L) (Fig. 1, Table 1). One month later, the patient was admitted to the emergency department for a gangrenous acute cholecystitis with a fluid collection near the right colic angle and peritoneal effusion. Empirical antimicrobial therapy based on piperacillin-tazobactam first (2 days) then on imipenem (2 days) was suboptimal despite low MIC to imipenem (≤0.25 mg/L) (Fig. 1). After blood cultures turned positive, CAZ-AVI was initiated for 14 days. This treatment, combined with surgical management, including replacement of the biliary prosthesis, led to clinical resolution. Nine months later, EC2-459 F3 was recovered from a new intra-abdominal sample. Surprisingly, this isolate did not produce any carbapenemase but displayed resistance to CAZ-AVI (MIC = 16 mg/L) (Table 1). A new CMY-42 variant was identified, CMY-219, carrying a single G156D substitution according to the structural alignment-based numbering of class C β-lactamases scheme (18) (Fig. S1). A third isolate, EC3-511J2, produced both OXA-484 and CMY-219 and was also categorized as resistant to CAZ-AVI (Table 1).

**TABLE 1:** Antimicrobial susceptibility testing of clinical isolates and transformants

	Clinical isolates			Transformants		
	*E. coli* EC1-399F8 OXA-484 + CMY-42	*E. coli* EC2-459F3 CMY-219	*E. coli* EC3-511J2 OXA-484 + CMY-219	*E. coli* pTOPO-CMY-2	*E. coli* pTOPO-CMY-42	*E. coli* pTOPO-CMY-219
Amoxicillin^*a*^	>256	>256	>256	>256	>256	32
Amoxicillin-clavulanate^*a*^	>256	>256	>256	>256	>256	24
Piperacillin^*a*^	>256	>256	>256	64	128	4
Piperacillin-tazobactam^*a*^	>256	32	>256	4	4	1.5
Cefoxitin^*a*^	>256	>256	>256	>256	>256	24
Cefotaxime^*a*^	>32	>32	>32	8	>32	2
Ceftazidime^*a*^	>256	>256	>256	64	>256	64
Ceftazidime-avibactam^*b*^	1	16	16	0.25	0.5	4
Ceftazidime^*a*^ on cloxacillin agar	NR^*c*^	NR	NR	0.38	1	24
Ceftazidime-avibactam^*a*^ on cloxacillin agar	NR	NR	NR	0.19	0.5	2
Cefepime^*b*^	4	1	2	0.12	0.5	0.12
Cefepime-enmetazobactam^*b*^	2	1	1	≤0.06	0.12	≤0.06
Ceftolozane-tazobactam^*b*^	>16	>16	>16	1	1.5	16
Aztreonam^*b*^	16	>16	>16	8	>16	2
Aztreonam-avibactam^*b*^	2	1	4	0.12	0.5	0.12
Cefiderocol^*b*^	1	2	2	0.06	0.25	0.25
Ertapenem^*b*^	2	≤0.06	4	≤0.06	0.12	≤0.06
Imipenem^*b*^	≤0.25	0.12	0.5	0.5	0.25	0.25
Imipenem-relebactam^*b*^	≤0.25	0.12	0.5	0.25	0.5	0.25
Meropenem^*b*^	≤0.25	≤0.06	0.25	≤0.06	≤0.06	≤0.06
Meropenem-vaborbactam^*b*^	≤0.25	≤0.06	0.25	≤0.06	≤0.06	≤0.06
Ciprofloxacin^*b*^	>2	>2	>2	NR	NR	NR
Levofloxacin^*b*^	>2	>2	>2	NR	NR	NR
Amikacin^*b*^	2	2	1	NR	NR	NR
Gentamicin^*b*^	>16	>16	>16	NR	NR	NR
Tigecycline^*b*^	0.5	≤0.06	0.25	NR	NR	NR
Eravacycline^*b*^	0.25	≤0.06	0.25	NR	NR	NR
Colistin^*b*^	0.5	0.5	0.5	NR	NR	NR

^*a*^ Indicates MIC measured using a diffusion gradient strip.

^*b*^ Indicates MIC measured using broth microdilution.

^*c*^ NR, not realized.

All three *E. coli* isolates belonged to ST410. SNP analysis supported within-patient clonality compared with all other OXA-484-producing ST410 sent to the F-NRC until December 2024 (*n* = 38) (Table S1). Among these 38 isolates, *bla*_CMY_ was frequent (*n* = 35). Of note, no mutation was identified in the PBP2 or PBP3 sequences between the three isolates. The ST410-associated PBP3 polymorphisms (YRIN insertion and I536L), known to confer decreased susceptibility to ceftazidime, cefepime, aztreonam, and cefiderocol, were present (19, 20).

A total of eight plasmid replicons were identified in the genomes: IncY, IncFI1–IncFII, IncX3, IncIγ, IncFIB and IncQ1, and ColKP3. Contig alignment of EC1-399F8 revealed a partial match with known IncIγ-type plasmids pL3452210II_4 carrying *bla*_CMY-42_ (GenBank accession number NZ_CP076531) and pMB7671_5 carrying *bla*_CMY-185_ (NZ_CP127853) from *E. coli* (Fig. S2).

To assess the contribution of CMY-219 to CAZ-AVI resistance, *bla*_CMY-2_, *bla*_CMY-42_, and *bla*_CMY-219_ were amplified with their native upstream region using the primers CMY-For (5′-AACACACTGATTGCGTCTGACG-3′) and CMY-Rev (5′-AAGGAGGCCCAATATCCTGG-3′), cloned into pCR-Blunt II-TOPO, and electroporated into *E. coli* TOP10. Transformants expressing CMY-219 displayed a fourfold increase (0.25–4.0 mg/L) in CAZ-AVI MICs compared to those expressing CMY-42 or CMY-2. Conversely, MICs to all other cephalosporins except ceftolozane–tazobactam and cefiderocol were significantly decreased (Table 1). Ceftazidime MICs on cloxacillin-supplemented agar were higher for CMY-219 (24 mg/L) than for CMY-2/CMY-42 transformants (Table 1).

Crude extract assays were used to compare CMY activity and inhibition properties of AVI and cloxacillin in pTOPO-CMY-2, CMY-42, and CMY-219 transformants, as previously described (21). Ceftazidime hydrolysis was very low for CMY-2 and CMY-42 and undetectable for CMY-219. Cephalothin was therefore used as a reporter substrate for IC_50_ determination. CMY-2 and CMY-42 displayed comparable hydrolytic activities toward cephalothin, with specific activities of 690 and 562 mU/mg, respectively, whereas activity was approximately 150-fold lower for CMY-219 (4 mU/mg). This finding is consistent with the lower MICs observed for most β-lactams in pTOPO-CMY-219 transformants. Avibactam IC_50_ values were 27-fold higher for CMY-219 than for CMY-42, indicating reduced inhibition (Table 2). Cloxacillin IC_50_ values were also markedly increased (4,000-fold) for CMY-219 versus comparators, consistent with the cloxacillin-agar phenotype. Both avibactam and cloxacillin exhibited reduced inhibitory activity against CMY-219 in accordance with the observed increase in MICs.

**TABLE 2:** Specific activity and IC_50_ for avibactam and cloxacillin using crude extracts containing selected β-lactamases

	Specific activity (mU/mg)	IC_50_ (nM)	
	Cephalothin	Avibactam	Cloxacillin
CMY-2^*a*^	690	320	5.3
CMY-42^*a*^	562	400	3.7
CMY-219^*a*^	4	9,900	22,000

^*a*^ The extracts were obtained from *E. coli *TOP10 transformants expressing CMY variant in pTOPO.

Analysis of read depth did not support major *bla*_CMY_ copy-number variation across clinical isolates (*bla*_CMY_/chromosomal gene ratio 1.0–1.6) (22). Promoter region revealed an identical genetic environment among the three isolates, suggesting that different levels of expression were not involved in CAZ-AVI resistance (Fig. S2).

This *in vivo* selection of CMY-219 is concerning because CAZ-AVI is a key option against OXA-48-like-producing Enterobacterales (1, 2). Notably, the cefepime–enmetazobactam combination remains active against these isolates, raising the question of its potential role as an alternative treatment for OXA-48-like Enterobacterales, possibly limiting avibactam-driven selective pressure and the emergence of CMY variants. Recently, a large-scale genomic analysis of 167,518 *E. coli* genomes from EnteroBase identified *bla*_CMY-42_ in 23.05% of ST410, 11.56% of ST167, and 5.90% of ST405 isolates (23). Worryingly, these successful clones also frequently harbor carbapenemase genes (mainly NDM-5, OXA-181, and OXA-48) and exhibit intrinsic reduced susceptibility to β-lactams due to PBP3 mutations (9).

Shropshire et al. reported that 48 days of CAZ-AVI treatment selected CMY-185 in an *E. coli* ST410 isolate (15). In their study, double or triple mutations in CMY-2, including N346Y, were required to reduce CAZ-AVI susceptibility (15). In contrast, a single G156D substitution in the H5 alpha helix of CMY-42 emerged after a single 14-day CAZ-AVI course. This represents the first description of a CAZ-AVI resistance-conferring mutation within this structural domain of CMY. Interestingly, CMY-219 lacks the N346 substitution shared by other CAZ-AVI-resistant CMY variants (CMY-172/CMY-178/CMY-185/CMY-192). It also retains all the other highly conserved residues already known to mediate avibactam binding (e.g., 4S, 67K, 120Q, 150Y, 152N, 315K, and 316T) supporting an alternative resistance route (17). *In silico* modeling using Chimera software suggested that introducing a bulky Asp at position 156 may perturb the local environment near the highly conserved Y150 in the active-site region. Concomitantly, the lower activity against several β-lactams suggests a functional trade-off between avibactam resistance and cephalosporin hydrolysis, as reported for CMY-185 with cephalothin (24). Further structural and kinetic work (inhibitor docking and acylation/deacylation parameters) will be required to define the molecular basis of G156D-mediated resistance.

## SUPPLEMENTAL MATERIAL

Supplemental figures aac.01335-25-s0001.pdf Figures S1 and S2.

Table S1 aac.01335-25-s0002.xlsx SNP matrix.

ASM does not own the copyrights to Supplemental Material that may be linked to, or accessed through, an article. The authors have granted ASM a non-exclusive, world-wide license to publish the Supplemental Material files. Please contact the corresponding author directly for reuse.
