# Supplementary material for: In vivo selection of CMY-219 conferring resistance to ceftazidime-avibactam in an OXA-484-producing E. coli ST410
Source: Antimicrob Agents Chemother. 2026 Feb 12;70(3):e01335-25. doi: 10.1128/aac.01335-25 (PMC12959149; doi:10.1128/aac.01335-25)
Supplement: Supplemental figures — Figures S1 and S2. [file aac.01335-25-s0001.pdf]

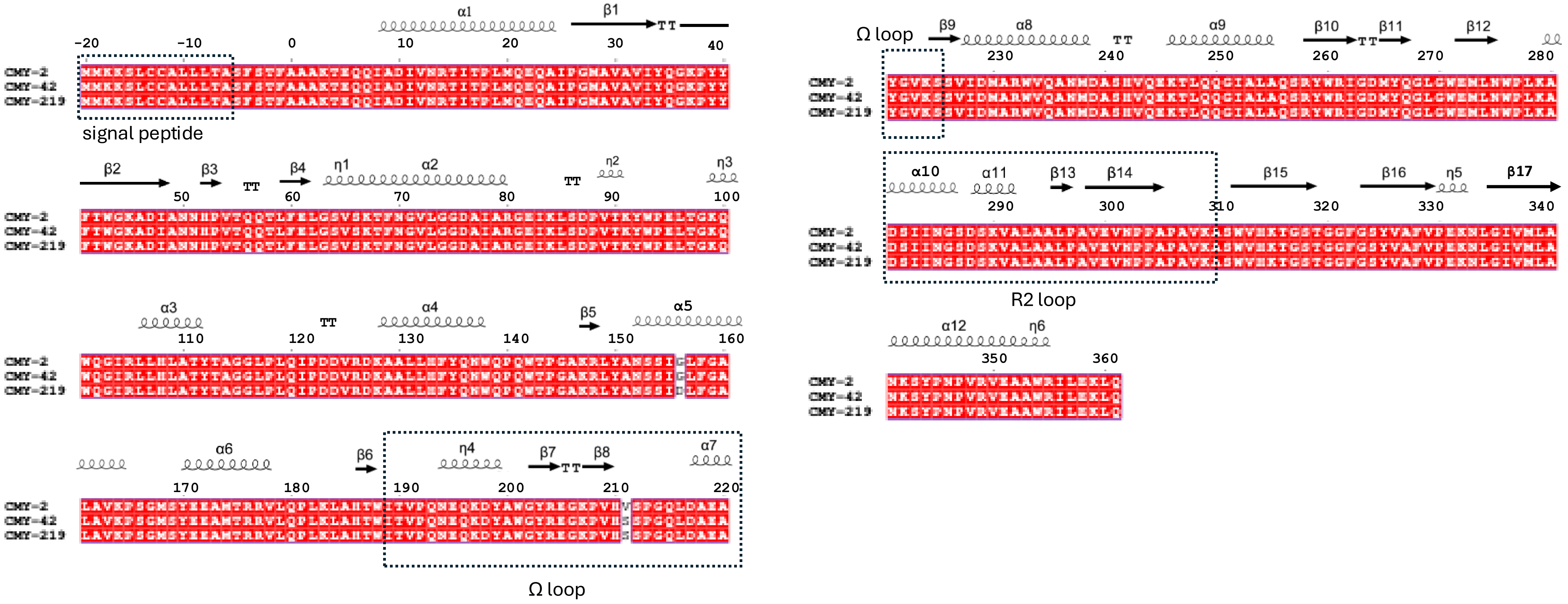

Figure S1. Amino acid sequence alignments of CMY-2, CMY-42 and CMY-219. The amino acid numbering of CMY was determined using the SANC (structural alignment-based numbering of class C β-lactamases) scheme. The sequence alignments of CMYs were generated by ClustalOmega and ESPript 3.0. The secondary structure of CMY-2 is used as PDB: 8DI7 and shown above the sequence, in which helices are squiggles, beta strands are arrows, and turns are TT letters

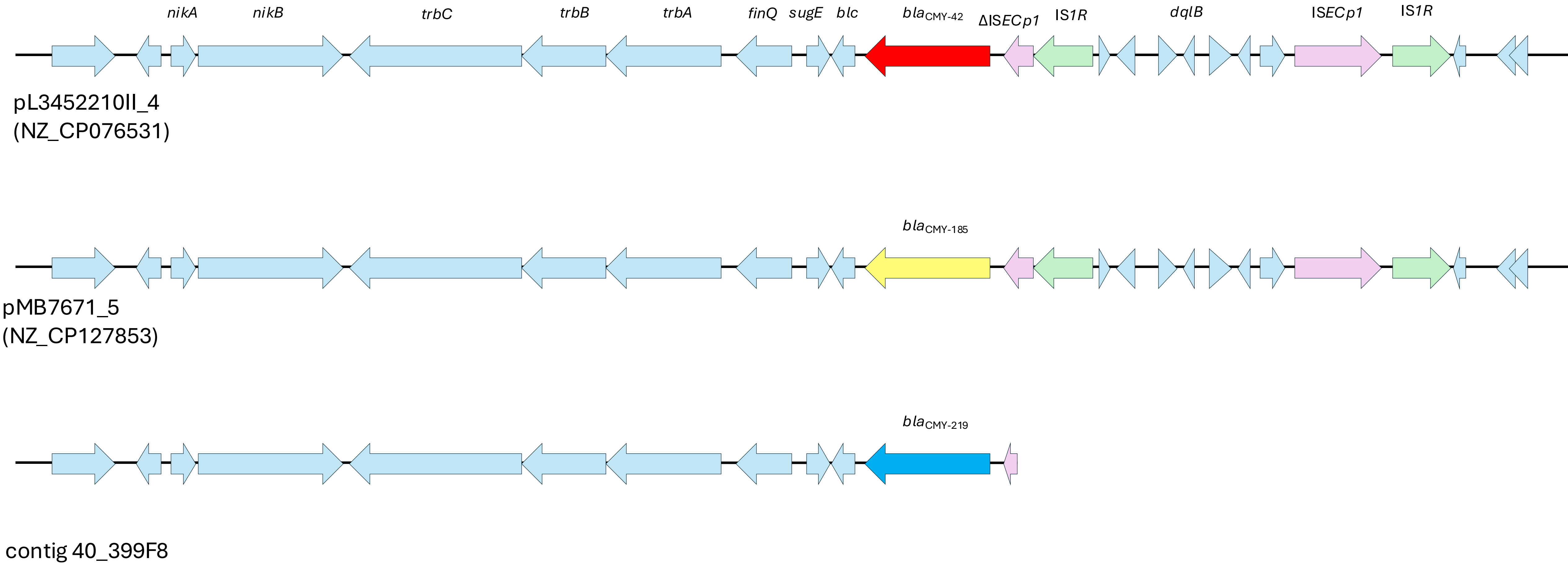

Figure S2. Zoom on the close genetic environment of *bla*$_{CMY}$ revealing structural similarity with previously sequenced plasmids of IncIγ type: pL3452210II_4 (carrying *bla*$_{CMY-42}$) and pMB7674_5 (carrying *bla*$_{CMY-185}$).
